# Determinants for a low health-related quality of life in asthmatics

**DOI:** 10.3109/03009734.2011.638730

**Published:** 2012-02-15

**Authors:** Mai Leander, Erik Lampa, Christer Janson, Kurt Svärdsudd, Monica Uddenfeldt, Anna Rask-Andersen

**Affiliations:** ^1^Department of Medical Sciences, Occupational and Environmental Medicine, Uppsala University, Uppsala, Sweden; ^2^The Red Cross University College, Department of Health Care Sciences, Stockholm, Sweden; ^3^Department of Medical Sciences, Respiratory Medicine and Allergology, Uppsala University, Uppsala, Sweden; ^4^Department of Public Health and Caring Sciences, Family Medicine and Clinical Epidemiology, Uppsala University, Uppsala, Sweden; ^5^Primary Care, County of Gävleborg, Sweden

**Keywords:** Asthma, GQL, generic instrument, quality of life

## Abstract

People with asthma suffer from impaired health-related quality of life (HRQL), but the determinants of HRQL among asthmatics are not completely understood. The aim of this investigation was to study determinants of low HRQL in asthmatics and to study whether the determinants of HRQL differ between sexes and age groups. A cohort of three age groups in Sweden was investigated in 1990 using a questionnaire with focus on respiratory symptoms. To study quality of life, the generic instrument Gothenburg Quality of Life was used. The participants were also investigated with interviews, spirometry, and allergy testing. Asthma was diagnosed in 616 subjects. Fifty-eight per cent (*n* = 359) of the subjects were women; and 24% were smokers, 22% ex-smokers, and 54% were non-smokers. Women were more likely than men to report poor health-related quality of life. Respiratory symptoms severity was another independent determinant of a lower quality of life as well as airway responsiveness to irritants. Current and former smokers also reported lower quality of life. Finally, absenteeism from school and work was associated with lower quality of life. Factors such as sex, smoking habits, airway responsiveness to irritants, respiratory symptom severity, allergy, and absenteeism from school and work were associated with low HRQL in asthmatics.

## Introduction

Health-related quality of life (HRQL) assessment is increasingly used as an outcome measure in asthma and other chronic respiratory diseases. Asthma is a common chronic disease worldwide, with an estimated 300 million affected individuals (1), and the prevalence of asthma in Sweden is about 10% (2). Asthma exerts a substantial adverse impact on the lives of many people with this condition (3). Although people with asthma suffer from impaired quality of life compared with people without asthma (4), the determinants of quality of life among people with clinically verified asthma have not been thoroughly explored (3). Reduced HRQL has been associated with female sex, young age, low lung function, low educational level, region of living, co-morbidity, use of inhaled bronchodilators and corticosteroids, wheezing, chronic cough, sputum production, and bronchial hyper-responsiveness (5,6). Our results from previous analyses of HRQL in asthmatics show that subjects with asthma had a higher level of non-respiratory symptoms and that subjects with low HRQL at baseline were more likely to develop asthma at follow-up 13 years later (7).

Understanding the determinants of quality of life in asthma, especially potentially modifiable ones, can be helpful in designing interventions that improve the quality of life in asthmatics (3). The primary aim of the present study was to study determinants of low HRQL in clinically verified asthmatics. A secondary aim was to study whether the determinants of HRQL were different in men and women as well as in younger and older asthmatics.

## Material and methods

### Study design and sample

**Figure 1.**
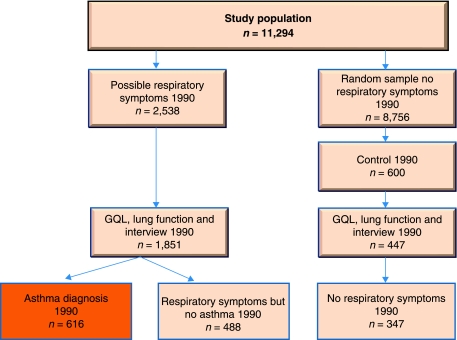
Study population and subgroups 1990–2003.

This study includes participants from two counties in Sweden: Jämtland and Gävleborg. In the 1990 cohort study, subjects were selected from the responders to a questionnaire focusing on respiratory symptoms sent to all individuals 16 years of age and to a random sample of the age groups of 30–39 and 60–69 years (8). A total of 11,294 subjects (89%) answered the questionnaire on respiratory symptoms. In a second phase, all subjects who reported a history of asthma, chronic bronchitis, or respiratory symptoms (*n* = 1,851) were invited to a clinical investigation including spirometry, allergy testing, and assessment of HRQL using the Gothenburg Quality of Life (GQL) questionnaire. The participation rate was 73%. In addition, a random sample of 600 subjects without a history of respiratory diseases and respiratory symptoms were invited to this phase of the investigation (8). The present analysis was based on 616 subjects with clinically verified asthma (Figure 1).

### Measurements

#### Questionnaire on respiratory symptoms

The postal questionnaire was based on the British Medical Research Council questionnaire, later modified and used in previous studies in northern Sweden (9,10). The questionnaire consisted of 22 items including questions on the following: attacks of dyspnoea, shortness of breath, or breathlessness; wheezing in the chest; prolonged productive cough; use of anti-asthmatic medication; and physician’s diagnosis of asthma, chronic bronchitis, or emphysema.

#### Gothenburg Quality of Life instrument

Subjective HRQL was measured in 1990 using the generic Gothenburg Quality of Life instrument (11). Part one of the instrument covers 30 common symptoms from various parts of the body; the respondents answer ‘yes’ or ‘no’ to questions on whether they had experienced any of the symptoms during the previous 3 months. The number of symptoms was summarized into a score. In order to be able to compare with the well-being scores below, the scale was transformed to a five-step score and inverted. Thus a score of five means no symptoms at all, and a score of zero means ‘yes’ to all the symptom questions.

Part two of the instrument focusing on well-being includes 18 items covering subjective physical, mental, and social well-being. The respondent answers each item on a seven-step Likert scale ranging from 1 = ‘very bad’, to 7 = ‘excellent/could not be better’. A high score therefore indicates a better level of well-being. An index was calculated for each of the three well-being scales by summarizing all items within each scale for each respondent and then dividing with the number of items within each scale (4). GQL has been validated using a Swedish population sample and by analysing biomedical variables (11).

#### Clinical measurements

Lung function was tested with pneumotachographs (Vitalograph Alpha, London, UK). Vital capacity (VC) and forced expiratory volume in 1 second (FEV_1_) were measured. This study adhered to the American Thoracic Society (ATS) (12) recommendations for spirometry testing. A skin prick test was performed using Phazet devices (Pharmacia Diagnostics, Uppsala, Sweden), which are stainless lancets pre-coated with extracts of ten common allergens (13). Lancets with allergen were applied on the skin inside the forearm. A skin test was said to be positive if the mean wheal diameter was 3 mm or more.

### Asthma diagnosis

Asthma was diagnosed if all of the following symptoms and findings were present (8): 1) repeated attacks of wheezing during the last year; 2) repeated attacks of dyspnoea with shortness of breath during the last year; 3) identified exogenous agents (at least three including allergens or unspecific factors) provoking shortness of breath and/or wheezing; 4) complete recovery between attacks; and 5) best FEV_1_ ≥80% of predicted.

If the history suggested asthma with repeated episodes of dyspnoea and/or wheezing in the last year and at least one positive answer to questions on wheezing or on long-standing dry cough, but did not meet the above criteria, the diagnosis was confirmed by at least one of the following observations: 1) reversibility of 15% from initial value in FEV_1_ after salbutamol inhalation; 2) variability in PEF (peak expiratory flow) ≥20% (amplitude % mean) 3 of 7 days; 3) PC20 ≤4 mg/mL or PD20 ≤2.6 mg (PC20 = provocative concentration of a substance (methacholine) causing a 20% fall in FEV_1_; PD20 = dose of histamine provoking a fall in FEV_1_ of 20%); and 5) best FEV_1_ ≥80% of predicted.

### Determinants

From the investigation in 1990 with respiratory questionnaires and interviews by a trained nurse the following questions were chosen in order to estimate determinants of asthma. The process of choosing these variables was based on discussions in the research team and also on earlier research and clinical experience.

#### Severity of respiratory symptoms

The severity of current obstructive symptoms was classified into four groups: no symptoms, wheeze during the last year, nightly symptoms such as wheezing or breathlessness or asthma attacks during the last year, and breathlessness during most days of the week.

#### Allergy tests

The subjects were graded into five groups depending of the results of the skin prick tests. Subjects with no reaction to either animal dander, pollen, mould, or mites were classified into the first group, while subjects with reactions to all four types of allergens were classified into the fifth group.

#### Airway responsiveness to irritants

Symptoms indicating bronchial hyper-responsiveness to irritants were classified into six groups depending on the answers to five questions about breathlessness on exhaustion in cold air during the winter, and wheezing or severe cough after exposure to dust, cigarette smoke, vehicle exhaust/air pollution, strong smells, or cold air. Subjects with no such symptoms were graded into group 1 while subjects answering ‘yes’ to all five questions were grouped to the sixth group.

#### Asthma medication

The asthma medication was graded into four groups depending on how heavy the medication was. The first group included subjects with no treatment, the second group subjects who were on bronchodilators, the third group subjects with inhalation steroids, and the fourth group subjects on oral steroids.

#### Emergency visits because of breathing problems

The first group included subjects who had never attended emergency medical care because of respiratory symptoms, the second group subjects with emergency visit to a physician, and the third group subjects who had been admitted to hospital because of respiratory symptoms.

#### Sick-leave caused by respiratory symptoms

Sick-leave caused by respiratory symptoms was graded into five groups depending on the number of days the subject had been absent from work or school because of respiratory symptoms in the last 12 months. In the first group the subjects had not been off work or school at all because of respiratory symptoms, in the second group the subjects had been off up to 8 days, in the third group 8–30 days, in the fourth group 30–90 days, and in the fifth group the subjects had been off work or school more than 90 days because of respiratory symptoms.

### Statistical analysis

Associations between the outcome variables (symptom score, physical well-being, mental well-being, and social well-being) and the explanatory variables were studied using multivariate linear regression. Although separate linear regression models fitted to each of the outcome variables would give similar point estimates and standard errors, the use of a multivariate model allowed us to perform a single test of each explanatory variable on all four outcomes simultaneously. This minimizes the risk of interpreting an effect on e.g. one of the outcome variables as significant when the overall test across all outcomes proved inconclusive. Prior to fitting the regression model, the symptom score was reversed and re-scaled to match the range of the other outcome variables so that a higher score represented fewer symptoms.

In an initial model fit, all categorical explanatory variables with more than two categories were entered as factors, yielding a separate effect estimate for each level except the reference level. We then performed a joint test of all categorical variables where our null hypothesis was that all categorical variables could be represented as an ordinal variable, i.e. as a linear term in the regression model. As the test was highly significant we proceeded to test which, if any, of the categorical variables could be modelled linearly choosing a *P* value cut-off of 0.2. The final model fit included the same pre-specified variables as the initial fit but with four variables modelled as categorical and the rest modelled linearly. We then proceeded by testing four pre-specified interactions: sex and age, each interacting with airway reactivity and symptoms, and wheeze. All interaction terms with *P* values ≤ 0.2 were kept in the model. Finally residuals from the model were assessed with respect to normality and homogeneity by visually inspecting normal quartile plots and plots of the residuals versus the fitted values as well as versus the explanatory variables.

Several of the variables contained missing values. To account for the missing data, multiple imputations of the missing values have been recommended in the literature (14). We used multivariate imputation using chained equations (15) to create 15 imputed data sets and included in the imputation model all four outcome variables and all potential explanatory variables.

Residuals from the model showed no gross deviations from normality when plotted against normal quartiles, although the residuals for the mental score outcome showed slightly heavy tails indicating slight left-skewness. Plots of the residuals versus fitted values and residuals versus the explanatory variables revealed no recognizable pattern, indicating that model assumptions may not have been severely violated.

Imputation, model fitting and the residual analysis were done using Stata version 11.

### Ethical approval

The study was approved by the Ethics Committee at the University of Umeå (§222, 1989-12-12) and the Ethics Committee, Faculty of Medicine, Uppsala (Dnr. 01–313).

## Results

The characteristics of the study sample in asthma subjects are presented in Table I. More than half of the members of all age-groups were women. The youngest age group had significantly more positive allergy tests, and the two youngest age groups also reported more hyper-responsiveness to irritants and allergic rhinitis. The oldest age groups had significantly more severe symptoms of asthma and lower FEV_1_. A higher asthma medication score was significantly more prevalent in the oldest age group. The smoking habits and use of oral tobacco differed significantly between the age groups, with more smokers among the 30–39-year-olds compared to the 16-year-olds. The younger age groups were significantly more absent from work and school. The oldest age group lived significantly more often in an apartment, but the 16-year-olds lived more often in a house and in a city. The symptom score and mental well-being did not differ between the age groups, but the oldest age group had lower physical well-being, and the younger age groups had lower social well-being.

**Table I.** The characteristics of the study sample (*n* (%) and mean ± SD).

	All (*n* = 616)	16 y (*n* = 160)	30–39 y (*n* = 233)	60–69 y (*n* = 223)	*P* value
Women	359 (58)	85 (53)	138 (59)	136 (61)	0.29
FEV_1_ % of predicted	93 ± 15	96 ± 3	94 ± 15	90 ± 17	< 0.05
Skin prick test reactivity					
None	293 (48)	53 (33)	72 (31)	168 (75)	< 0.001
1 type of allergen^a^	132 (21)	30 (19)	57 (25)	45 (20)	
2 types of allergens	124 (20)	46 (29)	72 (31)	6 (3)	
3 types of allergens	51 (8)	24 (15)	24 (10)	3 (1)	
4 types of allergens	15 (2)	7 (4)	7 (3)	1 (0)	
Airway responsiveness to irritants					
None	55 (9)	10 (6)	21 (9)	24 (11)	< 0.01
1 irritant	112 (18)	31 (19)	42 (18)	39 (17)	
2 irritants	144 (23)	38 (24)	56 (24)	50 (22)	
3 irritants	157 (26)	46 (29)	65 (28)	46 (21)	
4 irritants	108 (18)	27 (17)	39 (17)	42 (19)	
5 irritants	38 (16)	8 (5)	8 (3)	22 (10)	
Respiratory symptom severity					
None	57 (9)	15 (9)	22 (9)	20 (9)	< 0.01
Wheeze last year	90 (15)	27 (17)	43 (18)	20 (9)	
Nocturnal symptoms	402 (65)	108 (68)	148 (64)	146 (65)	
Daily symptoms	67 (11)	10 (6)	20 (9)	37 (17)	
Allergic rhinitis	231 (43)	75 (50)	107 (53)	49 (27)	< 0.001
Asthma medication					
None	324 (54)	65 (42)	119 (52)	140 (65)	< 0.001
Bronchodilators	87 (15)	36 (23)	32 (14)	19 (9)	
Inhaled corticosteroids	162 (27)	52 (34)	61 (27)	49 (23)	
Oral corticosteroids	24 (4)	2 (1)	15 (7)	7 (3)	
Smoking history					
Non-smokers	334 (54)	147 (92)	70 (30)	117 (52)	< 0.001
Ex-smokers	135 (22)	5 (3)	71 (31)	59 (26)	
Smokers	146 (24)	8 (5)	91 (39)	47 (21)	
Oral tobacco use	84 (14)	24 (15)	41 (18)	19 (9)	< 0.05
Emergency visits ever					
None	358 (59)	82 (52)	134 (58)	142 (64)	0.16
≥1 emergency department visit	128 (21)	36 (23)	53 (23)	39 (17)	
≥hospitalization	125 (20)	40 (25)	43 (19)	42 (19)	
Sick-leave during last 12 months					
None	526 (85)	118 (74)	197 (85)	211 (95)	< 0.001
1–8 days	37 (6)	20 (13)	16 (7)	1 (0)	
8–30 days	44 (7)	20 (13)	16 (7)	8 (4)	
30–90 days	5 (1)	1 (1)	3 (1)	1 (0)	
>90 days	4 (1)	1 (1)	1 (0)	2 (1)	
Living in an apartment	190 (33)	31 (21)	74 (32)	85 (43)	< 0.001
Living in a city	371 (64)	102 (69)	152 (67)	117 (57)	< 0.05
Health-related quality of life					
Symptom score	3.7 ± 1.3	4.0 ± 1.1	3.8 ± 1.4	3.7 ± 1.3	0.12
Social well-being	5.4 ± 0.9	5.4 ± 0.9	5.3 ± 0.9	5.5 ± 1.0	< 0.05
Physical well-being	4.2 ± 0.8	4.4 ± 0.8	4.3 ± 0.8	4.1 ± 0.8	< 0.001
Mental well-being	5.1 ± 1.0	5.2 ± 1.0	5.1 ± 1.1	5.1 ± 1.0	0.57

^a^Pollen, pets, mites, and mould.

**Figure 2.**
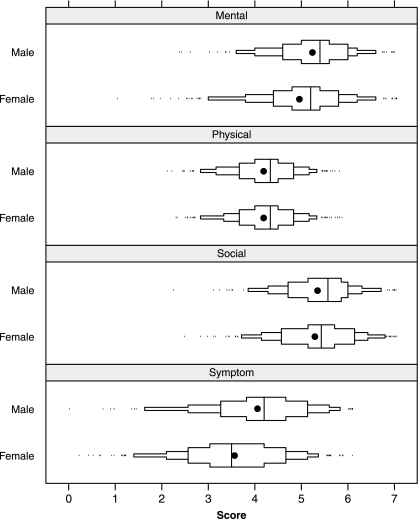
Distribution of the Gothenburg Quality of Life instrument subscales by sex. Solid dots indicate mean values, and solid line segments inside the boxes indicate the 25th, 50th, and 75th percentiles, respectively. Small ticks indicate observations outside the 5th and 95th percentiles. The *x*-axis represents the score on the seven-step Likert scale ranging from 1 = ‘very bad’, to 7 = ‘excellent/could not be better’.

Five determinants were independently related to impaired health-related quality of life in asthmatic subjects: female sex, hyper-responsiveness score, symptom severity, smoking habits, and absenteeism from work/school (Table II). Women were more likely than men to report poor health-related quality of life (Figure 2). Respiratory symptoms severity was another independent determinant of a lower quality of life as well as airway responsiveness to irritants (Table II). Current and former smokers also reported lower quality of life. Finally, absenteeism from school or work was associated with lower quality of life.

**Table II.** Determinants of asthma subjects. Multivariate linear regression, imputed data (*n* = 616). A negative value indicates lower quality of life. All coefficients for the score variables are per unit increase in the score unless noted otherwise.

	Symptom score		Social well-being		Physical well-being		Mental well-being		*P* value
Male sex	0.37	(0.14 to 0.60)	0.07	(–0.12 to 0.26)	–0.02	(–0.16 to 0.12)	0.22	(0.04 to 0.41)	< 0.001
Age group									
16 y	–0.05	(–0.34 to 0.24)	0.03	(–0.19 to 0.2)	–0.02	(–0.19 to 0.16)	–0.07	(–0.31 to 0.17)	0.102
30–39 y		0		0		0		0	
60–69 y	–0.08	(–0.36 to 0.19)	0.04	(–0.18 to 0.2)	–0.19	(–0.35 to –0.02)	0.01	(–0.21 to 0.23)	
Skin prick test reactivity score	0.16	(0.04 to 0.28)	0.03	(–0.06 to 0.13)	0.05	(–0.02 to 0.12)	0.10	(0.01 to 0.19)	0.059
Airway responsiveness to irritants									
None	0.47	(0.07 to 0.86)	–0.14	(–0.45 to 0.17)	0.18	(–0.05 to 0.42)	0.04	(–0.28 to 0.36)	< 0.001
1 irritant	0.59	(0.26 to 0.92)	0.35	(0.09 to 0.60)	0.43	(0.24 to 0.62)	0.37	(0.11 to 0.62)	
2 irritants	0.14	(–0.17 to 0.45)	0.17	(–0.05 to 0.40)	0.22	(0.05 to 0.40)	0.16	(–0.07 to 0.40)	
3 irritants		0		0		0		0	
4 irritants	–0.08	(–0.41 to 0.25)	0.11	(–0.14 to 0.36)	0.13	(–0.07 to 0.32)	0.05	(–0.22 to 0.32)	
5 irritants	–0.41	(–0.88 to 0.06)	0.24	(–0.14 to 0.62)	0.20	(–0.07 to 0.48)	0.20	(–0.17 to 0.58)	
Respiratory symptom severity score									
Females	–0.29	(–0.48 to –0.10)	–0.08	(–0.22 to 0.05)	–0.15	(–0.27 to –0.04)	–0.24	(–0.39 to –0.09)	0.187
Males	–0.27	(–0.47 to –0.06)	0.04	(–0.12 to 0.21)	–0.02	(–0.14 to 0.10)	0.00	(–0.16 to 0.17)	
Allergic rhinitis	–0.24	(–0.49 to 0.02)	–0.13	(–0.36 to 0.10)	–0.05	(–0.20 to 0.10)	–0.21	(–0.41 to –0.01)	0.169
Asthma medication score	0.10	(0.01 to 0.19)	0.05	(–0.03 to 0.12)	0.06	(0.00 to 0.11)	0.03	(–0.05 to 0.10)	0.154
Smoking habits									
Non-smokers		0		0		0		0	0.011
Ex-smokers	–0.11	(–0.39 to 0.17)	–0.14	(–0.35 to 0.07)	–0.28	(–0.45 to –0.11)	–0.25	(–0.49 to –0.02)	
Smokers	–0.36	(–0.65 to –0.08)	–0.24	(–0.47 to –0.01)	–0.27	(–0.44 to –0.10)	–0.34	(–0.57 to –0.11)	
Oral tobacco use	0.03	(0.32 to 0.38)	0.27	(0.03 to 0.52)	0.15	(–0.05 to 0.34)	0.13	(–0.13 to 0.39)	0.188
Sick-leave during last 12 months									
No sick leave		0		0		0		0	0.025
<8 days	–0.23	(–0.65 to 0.20)	–0.06	(–0.38 to 0.27)	–0.01	(–0.26 to 0.25)	–0.14	(–0.49 to 0.21)	
8–30 days	–0.81	(–1.21 to –0.41)	–0.24	(–0.53 to 0.06)	–0.24	(–0.47 to 0.00)	–0.44	(–0.76 to –0.11)	
30–90 days	–1.13	(–2.20 to –0.07)	–0.28	(–1.14 to 0.59)	0.08	(–0.58 to 0.75)	–0.51	(–1.41 to 0.40)	
>90 days	–1.23	(–2.42 to –0.04)	–0.56	(–1.60 to 0.49)	–0.39	(–1.13 to 0.34)	–0.98	(–1.98 to 0.03)	

The result of an interaction analysis indicated (*P* = 0.19) that a higher symptom score had more impact in women and to a certain degree also mental and social well-being, while symptom score seemed to have quite similar effects on the physical well-being between the sexes.

No significant interaction was found between age and symptom severity (*P* = 0.89). Neither age nor gender seemed to modify the effect of hyper-responsiveness to irritants on quality of life (*P* = 0.82 and 0.88, respectively).

## Discussion

In this cross-sectional study of determinants of quality of life in people with clinically verified asthma, the results show that female sex, smoking habits, higher airway responsiveness to irritants, respiratory symptom severity, positive skin prick test, and absenteeism from work/school were significantly associated with health-related quality of life.

To our knowledge this is the first study of determinants of quality of life in asthmatics using the Gothenburg Quality of Life instrument. One finding of this study is that women with asthma report poorer HRQL than men. Several other studies have also found that women with asthma experience greater impairment of the health-related quality of life (5,6,16) despite the fact that women had, on average, a higher lung function than men. The explanation could be that women have a poorer overall health compared to men, additional co-morbidities contributing to the HRQL impact, or that women have a more severe asthma disease in terms of subjective disease parameters. Asthma in women has been hypothesized to be related to natural changes in sex-steroid hormone levels during a woman’s life, which might be an explanation. In particular, women’s airways are the subject of cyclical variations of sex hormones that occur in relation to circadian rhythms, menstrual cycles, hormonal contraceptive use, pregnancy, and menopause (17).

It has been suggested that women are more emotionally distressed by the sheer presence of asthma symptoms, while men do not react until their symptoms become severe and long-standing (16). Osborne and co-workers also reported that women had more symptoms and poorer HRQL than men. They suggested that the differences could be related to the response to the disease rather than real differences in the disease between men and women. In a recent review on sex differences in asthma it was also noted that women with asthma report a greater number of unscheduled physician visits, more frequent use of oral corticosteroids, and a greater use of short-acting beta-agonists for rescues than men. Understanding gender differences in response to chronic disease such as asthma is important in tailoring management plans to each individual patient (18). Physicians should think about that men may have poor pulmonary function levels, despite the fact that they may not be bothered by their disease, while women’s complaints concerning their asthma should be taken seriously (19).

Current and former smokers reported lower health-related quality of life in the present study. A previous study shows that asthmatics who were current smokers had lower lung function and more co-morbidity, wheezing, and hyper-responsiveness than non-smoking asthmatics (6). Cigarette smoking appears to be associated with more asthma symptoms in adults. Smoking is also a life-style factor, and efforts to assist people with asthma who smoke to change this behaviour may help to improve their quality of life (3). Our results are in line with a study showing that smokers have a greater risk for severe asthma than non-smokers (20).

In the present study, respiratory symptom severity was also found to be a determinant of low HRQL, but a higher number of symptoms also seems to have a worse impact on mental well-being in women than in men. This is in accordance with other studies showing that low HRQL was primarily related to asthma symptoms (6) and with another study showing that there was a relationship between respiratory symptoms and anxiety as well as depression (21). In contrast, asthma was not found to be related to anxiety and depression in a population study (22).

The youngest age group had positive allergy tests and higher airway responsiveness to irritants significantly more often than the two older age groups. The social well-being in the youngest age group was significantly lower than in the two older age groups, but the physical well-being was significantly higher. In contrast, another study found that the physical well-being was lower in the younger age group (16). Allergic rhinitis remains a significant health problem because of the high burden of symptoms and its impact on general well-being and HRQL (23). A possible explanation for the low social well-being in the youngest age group can be that adolescents and younger children are in a developmental stage of their life and are therefore struggling with several issues that can be difficult to handle due to changes in the pattern of the disease and also the demands of everyday life (24).

The oldest age group had significantly more symptoms, lower lung function, and used more asthma medication. They had a significantly poorer physical well-being score than the two younger age groups. Ford and co-workers demonstrated clearly that asthma significantly affected HRQL among the elderly (3). Furthermore, Larsson and co-workers reported that older women with asthma reported the worst HRQL impairments (16). In general, studies have found that quality of life in asthma is worse among older patients than among younger patients (25). Among the elderly, co-morbidities probably account for some of the associations with poor or fair health. However, in this study diseases other than asthma were excluded in the analysis.

Absenteeism from work and school was associated with lower quality of life. Ford and co-workers found that inability to work was strongly associated with impaired HRQL (3). A plausible explanation for this association is that subjects are unable to work or go to school because of their asthma and this contributed to lower HRQL. There can also be life-style and socio-economic factors that add to the complexities of this issue. Unfortunately this study did not include such variables.

The strength of this study is the clinically verified asthma diagnosis and the validated quality of life instrument, but the results are also subject to some limitations. As this was a cross-sectional study, the cause and effect of these associations cannot be determined. Another limitation is the lack of socio-economic classification, but type of dwelling (apartment or house) as well as living in the city or country-side were used together as a surrogate marker. Unfortunately, weight was not registered. Despite this, we believe that our results contribute to the knowledge of quality of life in asthma.

In conclusion, this study identified factors associated with poor HRQL in people with clinically verified asthma. The results suggest that a number of factors such as sex, smoking habits, airway responsiveness to irritants, respiratory symptom severity, allergy, and absenteeism from school and work are associated with HRQL in asthmatics. Women with asthma report lower HRQL than men. A better understanding of the factors associated with HRQL in asthma is important in order to improve asthma management.
